# Patterns of traumatic outdoor rock-climbing injuries in Sweden between 2008 and 2019

**DOI:** 10.1186/s40634-021-00407-1

**Published:** 2021-10-09

**Authors:** Fredrik Identeg, Ebba Orava, Mikael Sansone, Jon Karlsson, Henrik Hedelin

**Affiliations:** Orthopaedic department, Institute of Clinical Sciences, Sahlgrenska Academy, University of Gothenburg, Gothenburg, Sweden

**Keywords:** Rock climbing, Climbing-related-injury, Climbing, Trauma, Sports injuries

## Abstract

**Purpose:**

Injury prevalence patterns for climbers have been presented in several papers but results are heterogenous largely due to a mix of included climbing disciplines and injury mechanisms. This study describes the distribution and pattern of acute traumatic climbing injuries sustained during outdoor climbing in Sweden.

**Methods:**

Patients that experienced a climbing related traumatic injury during outdoor climbing between 2008 and 2019 and who submitted a self-reported questionnaire to the Swedish Climbing Association were included in the study. Medical records were retrieved, and the International Climbing and Mountaineering Federation injury classification system was used for injury presentation.

**Results:**

Thirty-eight patients were included in the study. Seven (18%) injuries occurred during traditional climbing, 13 (34%) during sport climbing and 9 (24%) during bouldering. Varying with climbing discipline, 84–100% injuries were caused by falls. Injuries of the foot and ankle accounted for 72–100% of the injuries. Fractures were the most common injury (60%) followed by sprains (17%) and contusions (10%).

**Conclusions:**

Traumatic injuries sustained during outdoor climbing in Sweden were predominantly caused by falls and affected the lower extremities in all major outdoor climbing disciplines. Rope management errors as a cause of injury were common in sport climbing and in activity surrounding the climbing, indicating there is room for injury-preventing measures.

## Background

Over the last decades, rock climbing has continuously gained popularity as a recreational sport, with the number of participants increasing in recent years. In Sweden, around 13,000 individuals were affiliated with a climbing club in 2018, with the number of people climbing regularly likely to be significantly higher [1].

Rock climbing consists of several different disciplines with distinct patterns of injury [2]. Outdoor sport climbing use dynamic ropes running through pre-set bolts to absorb the force when a fall occurs, while in traditional climbing, the roped climber relies on temporarily affixed removable protection. In bouldering, the climber ascends boulders of varying height, using padded mats to reduce ground reaction forces when landing.

Outdoor climbing in Sweden mainly take place on compact granite rock. The majority of rock-climbing routes in Sweden are non-bolted traditional climbs with crack systems of good rock-quality. Loose rock is at times present on less frequently climbed routes. In sport climbing, recently bolted routes tend to have a safe distance (1–3 m) between the bolts while routes set up in the 1990’s adhered to the more restrictive bolting policies of that time and thus have longer potential falls. Bolts are usually fixed in the rock through self-expanding bolts, with the exception of a minority of bolts anchored through liquid resin. Bolt failure is uncommon.

The distribution of climbing related injuries across climbing disciplines include a wide spectrum of injuries ranging from chronic overuse injuries, acute strains, and ligament tears to severe high energy polytraumas, occurring mostly from falls [3–5]. Although indoor climbing is far more commonly practiced when compared to outdoor climbing, outdoor climbing seems to pose a higher injury risk than indoor climbing, when analyzing injury rate per 1000 h, as suggested in a 2010 review by *Schöffl* et al [2]. Injury distribution also varies with climbing discipline, and studies examining the injury pattern of outdoor climbing in general, show varying results, with several studies suggesting a higher injury rate of the upper extremities, [6, 7] while other studies point towards a higher injury risk of the lower extremities [4, 8–11]. Examining each outdoor climbing discipline separately, traditional climbing seems to pose a higher risk of injury compared to sport climbing and bouldering [5, 12]. Injuries in traditional climbing also tend to be traumatic injuries mainly caused by falls, while sport climbing injuries are more commonly a result of an acute overuse [12, 13]. Outdoor bouldering injuries usually consist of both traumatic injuries and acute overuse.

Although, a broad body of literature have been published on the epidemiology of climbing-related injury, studies reporting injuries individually for each climbing-discipline while also separating between outdoor climbing and indoor climbing are scarce [7, 8, 12, 14–17]. While large samples from e.g., Emergency departments have been presented, providing general understanding of the patterns of traumatic injuries in rock-climbing, detailed descriptions regarding the nature of traumatic injuries in each outdoor climbing discipline is lacking [18–21].. A systematic review by Rauch et al. in 2019 on climbing accidents associated with acute injuries, concluded the need for further studies presenting injury data for each subdiscipline of climbing to enhance the understanding of the injury patterns of the sport [19].

In Sweden, the only previous study describing climbing related injuries, was presented by *Backe* et al. in 2012, and investigated all climbing-related injuries among a sample of members of the Swedish Climbing Association using self-reported questionnaires (*n* = 355, 106 injuries) [8]. Injuries in this study consisted almost exclusively of overuse injuries (93%). Detailed descriptions of traumatic outdoor climbing injuries in Sweden are to date, lacking.

The aim of this study is to describe the distribution, and nature of acute traumatic climbing injuries sustained during outdoor climbing from 2008 to 2019 in Sweden. Injury patterns, mechanisms of injury as well as the cause of injury are described for each climbing discipline.

## Materials and methods

### Patients

Since 2008, The Swedish Climbing Association (SKF) host a web-based register, available at all times through SKF’s webpage, where climbers in Sweden are to report all climbing related accidents and near-accidents [22]. Climbers are regularly (annually) encouraged to report injuries occurring through information- and news-platforms of the SKF. The self-reported questionnaire contains demographic questions regarding involved climbers, belayers, the traumatic event and the injuries sustained while climbing, and have previously been used primarily by the SKF to analyze technical errors and to implement safety measures.

Climbers who answered the questionnaire of the SKF’s web-based register, reporting an incident where an injury occurred during outdoor rock climbing between the years of 2008–2019 were included in the study. Patients were contacted through e-mail for consent regarding inclusion in the study.

Injuries sustained during traditional climbing, sport climbing, bouldering and self-belayed climbing were included. Climbers who sustained an injury during activities surrounding the climbing activity, (ascending/descending a climbing crag/boulder, rappelling etc.) were also included in the study. Incidents occurring outside of Sweden, and climbers below the age of 18 at the time of inclusion were excluded, since the injury patterns of adolescents were outside the scope of this paper. Injuries that resulted in fatality were registered by the climbing-partner of the deceased.

Information regarding age, sex, and level of experience was retrieved from the questionnaires. Climbing sub-discipline and difficulty level of the route climbed when the injury occurred was recorded. The questionnaire included questions regarding both the course of the event that led to the injury (mechanism of injury), and a self-reported explanation of what caused the injury. To determine the mechanism and cause of injury, the described course of events included in the self-report questionnaire were read, interpreted, and classified by the authors.

### Injuries

A traumatic acute injury was defined as an injury occurring as a result of external forces (falls, rock falls etc.). Acute Injuries sustained as a result of internal forces (i.e. pulling on a grip) were not included. All injuries in regard to diagnosis and severity of injury was confirmed through medical records retrieved from the visited medical institution. Medical records were retrieved from visits to orthopedic departments and primary care after individual consent from each participant. The medical reports were filed by residents and specialists in orthopedic surgery and general medicine. All fractures were confirmed through radiographic imaging appropriate for the type of injury. The International Mountaineering and Climbing Federation (UIAA) medical committee classification system of injury was used to classify injury location, severity and outcome [23].

### Climbing grades

Grades of each route were converted into UIAA metric in accordance with the conversion scale of *Draper* et al [24]. The Swedish grade scale employed in traditional climbing is not included in any available research literature, and thus conversion was based on existing subject-specific literature [25].

### Statistical analysis

Descriptive analysis of data was calculated through IBM SPSS Statistics for MAC, version 24 (IBM Corp., Armonk, N.Y., USA. Normally distributed variables were reported as means with standard deviations (SD).

### Ethical approval

This study was approved by the Regional ethics committee (IRB) of Västra Götalandsregionen 2019-03-16, dnr 2019–00656.

## Results

**Fig. 1:**
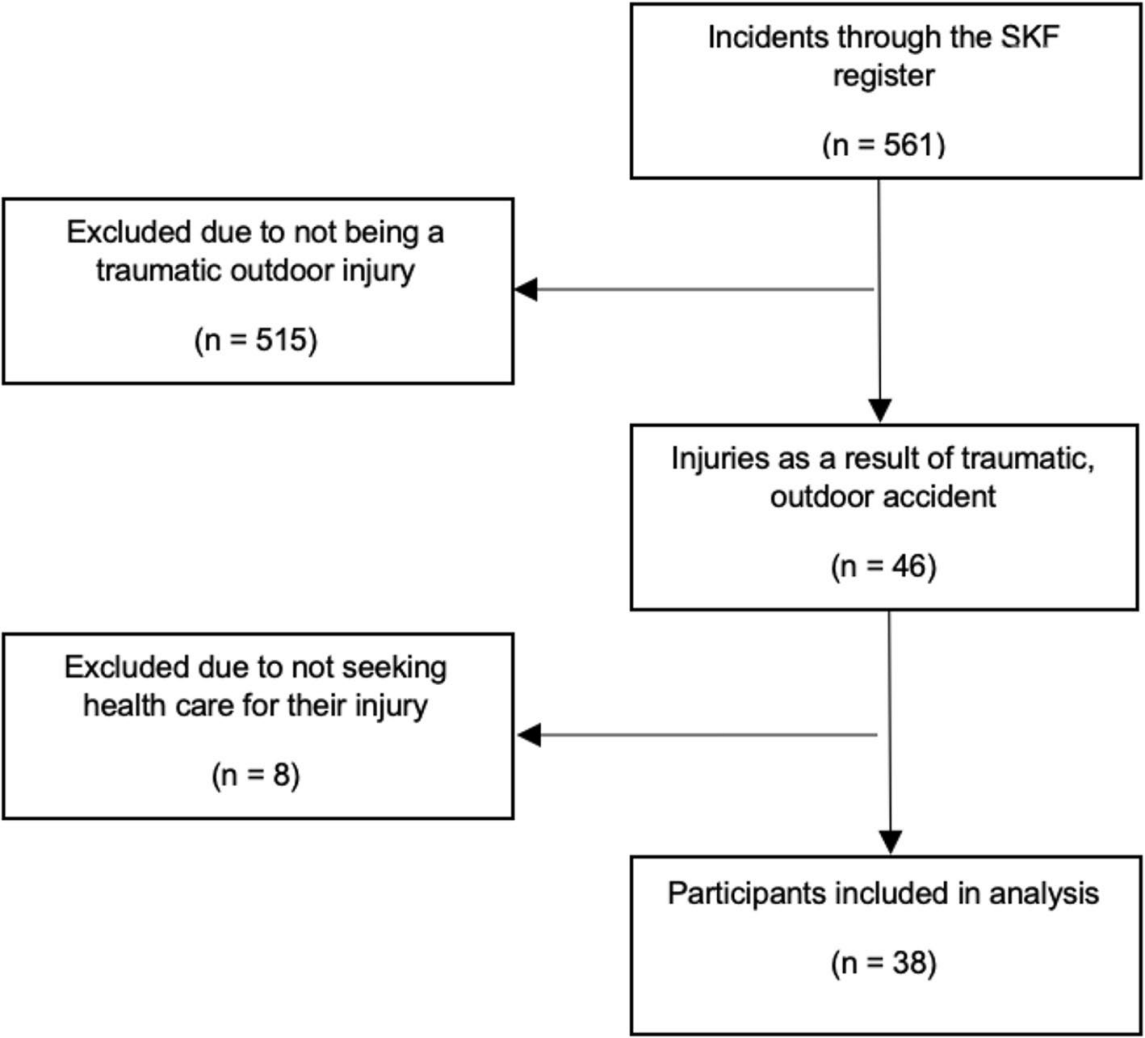
Flowchart of included participants

A total of 561 patients had completed the questionnaire of the SKFs’, web-based register. After application of the exclusion criteria, 46 participants were eligible for inclusion in the study. Eight patients were excluded due to not seeking healthcare for their injury (Fig. 1).

Of a total of 38 climbers included in the analysis, 30 participants were male and 8 female (Table 1)*.* The mean age of the participants at the time of the injury was 34 (SD 10) years old and the average climbing experience was 8.8 (SD 9.6) years. A total of 7 (18%) injuries occurred during traditional climbing, 13 (34%) during sport climbing, 9 (24%) during bouldering, 3 injuries (8%) occurred during top roping/self-belaying and six injuries (16%) in activities connected to the climbing (Table 1).

**Table 1:** Demographics of included participants

		Sex					
		Female		Male		Total	
		Mean (SD)	n (%)	Mean (SD)	n (%)	Mean (SD)	n (%)
Age		30 (±9.6)		35 (±11)		34 (±10)	
Age	< 25		2 (25)		3 (10)		5 (13)
	25–40		5 (63)		21 (70)		26 (68)
	> 40		1 (12)		6 (20)		7 (19)
	Total		8 (100)		30 (100)		38 (100)
Years climbing		6.1 (±4.3)		9.5 (±10.6)		8.8 (±9.6)	
Years climbing	< 5		5 (63)		15 (51)		20 (53)
	5–15		3 (37)		11 (37)		14 (37)
	> 15		0 (0)		3 (9)		3 (7)
	Missing		0 (0)		1 (3)		1 (3)
	Total		8 (100)		30 (100)		38 (100)
Experience	Beginner		2 (25)		6 (21)		8 (21)
	Experienced		4 (50)		11 (38)		15 (40)
	Very experienced		2 (25)		12 (41)		14 (37)
	Missing		0 (0)		1 (0)		1 (2)
	Total		8 (100)		30 (100)		38 (100)
Climbing type when injured	Bouldering		3 (38)		6 (20)		9 (24)
	Sport climbing		3 (38)		10 (33)		13 (34)
	Traditional climbing		0 (0)		7 (23)		7 (18)
	Surrounding activity		1 (12)		5 (17)		6 (16)
	Top rope/clogging		1 (12)		2 (7)		3 (8)
	Total		8 (100)		30 (100)		38 (100)

Of all injuries, a total of 68% were in the lower extremities while 19% of the injuries were of the spine, trunk pelvis and buttocks. Five percent of all injuries were in the upper extremities (Table 2). Fractures were the most common injury (60%) followed by sprains (17%) and contusions (10%). Fractures, sprains and contusions of the foot and ankle accounted for 66% of all injuries (Table 3). The injury severity according to the UIAA severity score was 1, 24%, 2, 39%, 3, 32%, 4, 0% and 5, 5% (fatality).

**Table 2:** Injury location, injury severity and type of injury for each climbing discipline

		Bouldering	Sport climbing	Traditional climbing	Surrounding activity	Top rope/ self-belaying	Total
		n (%)	n (%)	n (%)	n (%)	n (%)	n (%)
Injury location	Upper extremities	0 (0)	1 (8)	0 (0)	0 (0)	1 (33)	2 (5)
	Lower extremities	9 (100)	11 (84)	5 (72)	0 (0)	1 (33)	26 (68)
	Head	0 (0)	0 (0)	1 (14)	0 (0)	0 (0)	1 (3)
	Spine	0 (0)	0 (0)	1 (14)	2 (33)	0 (0)	3 (8)
	trunk, pelvis and buttock	0 (0)	1 (8)	0 (0)	2 (33)	1 (34)	4 (11)
	Location unspecified	0 (0)	0 (0)	0 (0)	2 (34)	0 (0)	2 (5)
	Total	9 (100)	13 (100)	7 (100)	6 (100)	3 (100)	38 (100)
Detailed Injury location	Head and neck	0 (0)	0 (0)	1 (14)	0 (0)	0 (0)	1 (3)
	Upper limbs/shoulder/clavicle	0 (0)	1 (8)	0 (0)	0 (0)	1 (33)	2 (5)
	Trunk, chest (sternum/ribs)	0 (0)	1 (8)	0 (0)	1 (17)	0 (0)	2 (5)
	Thoracic spine	0 (0)	0 (0)	1 (14)	0 (0)	0 (0)	1 (3)
	Lumbar spine	0 (0)	0 (0)	0 (0)	2 (33)	0 (0)	2 (5)
	Pelvis, buttock	0 (0)	0 (0)	0 (0)	1 (17)	1 (33)	2 (5)
	Knee	0 (0)	1 (8)	0 (0)	0 (0)	0 (0)	1 (3)
	Ankle	6 (67)	6 (46)	4 (58)	0 (0)	1 (34)	17 (45)
	Foot	3 (33)	4 (30)	1 (14)	0 (0)	0 (0)	8 (21)
	Location unspecified	0 (0)	0 (0)	0 (0)	2 (33)	0 (0)	2 (5)
	Total	9 (100)	13 (100)	7 (100)	6 (100)	3 (100)	38 (100)
Type of injury	Fracture	8 (89)	8 (61)	3 (43)	4 (67)	0 (0)	23 (60)
	Sprain	1 (11)	3 (23)	2 (29)	0 (0)	0 (0)	6 (17)
	Contusion	0 (0)	1 (8)	1 (14)	0 (0)	2 (67)	4 (10)
	Tendon rupture	0 (0)	1 (8)	0 (0)	0 (0)	1 (33)	2 (5)
	Commotio	0 (0)	0 (0)	1 (14)	0 (0)	0 (0)	1 (3)
	Fatal injury	0 (0)	0 (0)	0 (0)	2 (33)	0 (0)	2 (5)
	Total	9 (100)	13 (100)	7 (100)	6 (100)	3 (100)	38 (100)
UIAA Illness and Injury Severity Score	1	1 (11)	4 (31)	2 (29)	0 (0)	2 (67)	9 (24)
	2	4 (45)	6 (46)	3 (42)	1 (17)	1 (33)	15 (39)
	3	4 (44)	3 (23)	2 (29)	3 (50)	0 (0)	12 (32)
	4	0 (0)	0 (0)	0 (0)	0 (0)	0 (0)	0 (0)
	5	0 (0)	0 (0)	0 (0)	2 (33)	0 (0)	2 (5)
	Total	9 (100)	13 (100)	7 (100)	6 (100)	3 (100)	38 (100)

**Table 3:** Injury location of fractures, sprains and contusions

		Fracture	Sprain	Contusion	Total
		n (%)	n (%)	n (%)	n (%)
Injury location UIAA	Upper limbs/shoulder/clavicle	0 (0)	0 (0)	1 (25)	1 (3)
	Trunk, chest (sternum/ribs)	2 (9)	0 (0)	0 (0)	2 (6)
	Thoracic spine	1 (4)	0 (0)	0 (0)	1 (3)
	Lumbar spine	2 (9)	0 (0)	0 (0)	2 (6)
	Pelvis, buttock	1 (4)	0 (0)	1 (25)	2 (6)
	Ankle	10 (44)	6 (100)	1 (25)	17 (52)
	Foot	7 (30)	0 (0)	1 (25)	8 (24)
	Total	23 (100)	6 (100)	4 (100)	33 (100)

In bouldering, 89% of the injuries were sustained during a fall (Table 4). One hundred percent of the injuries were in the foot and ankle and 89% of all injuries were fractures.

**Table 4:** Mechanism of injury and cause of incident for each climbing discipline

		Bouldering		Sport climbing		Traditional climbing		Surrounding activity	Top rope/ clogging		Total	
		n (%)	Mean (SD)	n (%)	Mean (SD)	n (%)	Mean (SD)	n (%)	N (%)	Mean (SD)	n (%)	Mean (SD)
Mechanism of injury	Roped fall	0 (0)		11 (84)		7 (100)		6 (100)	3 (100)		27 (71)	
	Bouldering fall	8 (89)		0 (0)		0 (0)		0 (0)	0 (0)		8 (21)	
	Struck by falling object/person	0 (0)		1 (8)		0 (0)		0 (0)	0 (0)		1 (3)	
	Other mechanism of injury	1 (11)		1 (8)		0 (0)		0 (0)	0 (0)		2 (5)	
	Total	9 (100)		13 (100)		7 (100)		6 (100)	3 (100)		38 (100)	
Cause of incident	Error rope management climber	0 (0)		0 (0)		0 (0)		5 (83)	0 (0)		5 (13)	
	Grip falls off	1 (11)		0 (0)		1 (14)		0 (0)	0 (0)		2 (5)	
	Fall	8 (89)		9 (69)		6 (86)		1 (17)	3 (100)		27 (71)	
	Error rope management belayer	0 (0)		4 (31)		0 (0)		0 (0)	0 (0)		4 (11)	
	Total	9 (100)		13 (100)		7 (100)		6 (100)	3(100)		38 (100)	
Grade UIAA metric			8.0 (±0.9)		7.3 (±1.1)		6.8 (±1)			6.2 (±0.2)		7.0 (±1.2)

In sport climbing, 84% of all injuries were the result of a fall (Table 4), and 76% of all injuries were of the foot and ankle. Sixty-one percent of all injuries were fractures. Thirty-one percent of the injuries sustained during sport-climbing were reported to be caused by rope error management by the belayer.

In traditional climbing, 100% of the injuries were sustained during a fall (Table 4) and 72% of the injuries were of the foot and ankle. Forty-three percent of all injuries were fractures.

Of the injuries sustained surrounding the climbing activity, 100% of the injuries were the result of a fall, and 33% of these resulted in fatality. 83% of the injuries sustained surrounding the climbing activity were caused by rope management errors by the injured (Table 4).

## Discussion

The aim of this study was to describe the distribution of acute traumatic climbing injuries sustained during each discipline of outdoor rock climbing in Sweden. The main result of this study was that traumatic outdoor climbing injuries in all the major rock-climbing disciplines (sport climbing, bouldering, and traditional climbing) were mainly caused by falls (84–100%) and predominantly affected the foot and ankle (72–100%).

As mentioned, a systematic review by Rauch et al. [19] examining the state of the literature on acute climbing injuries reported on the heterogeneity across previous studies regarding study design and presentation. Thus, comparing the results to previous studies is difficult on a subdiscipline-level. While the present study provides additional, complementary information regarding the more severe injuries prevalent in outdoor rock-climbing for each climbing discipline, the results also confirm of previous studies regarding both injury pattern and injury mechanism in of acute, traumatic climbing injuries in general. For example, two studies examining the injury patterns of people visiting emergency departments in the U. S, reported a high rate of falls, as well as foot and ankle injuries to be common injury locations [4, 10]. These studies examined injuries of both indoor and outdoor climbing.

Regarding injury severity, our results show a high rate of severe injuries (37% UIAA grade ≥ 3) compared to previous studies [6, 10]. In a study by *Buzzacott* et al*,* examining climbing related injuries of United States emergency department visits 12.7% of the injuries were of UIAA grade 3 or above [10]. Another study, by *Neuhof* et al evaluating injury risk and injury rate of sport climbing, found 18.6% of all injuries to be UIAA grade 3 or higher [6]. Similar to injury location, these previous studies, have however presented injuries sustained during both indoor climbing and outdoor climbing mixed, which can be expected to affect the results, since outdoor climbing injuries commonly lead to a higher injury severity compared to indoor climbing injuries [2]. The high prevalence of falls in this study may of course also be a contributing factor to the high injury severity of the present study. Over-reporting of more serious injury in the present study may also account for the discrepancy.

Furthermore, the results of this study showed individual mistakes to be the cause of 31% of the incidents in sport-climbing. Increased awareness of, and adherence to basic safety norms would therefore likely reduce the risk of traumatic injuries. Similarly, rope management errors by the person injured during activity surrounding climbing (such as repelling etc) shows the need for increased safety education.

The major strengths of this study include the clear focus on traumatic outdoor injuries occurring in rock climbing, presented separately for each climbing-discipline which provides new insights regarding the injury patterns of the sport. Other strengths include a well-defined sample of exclusively traumatic injuries with injury diagnosis confirmed through medical journals. The results in terms of type, mechanism and cause of injury provide useful information for implementation of injury prevention measures of traumatic climbing injuries. The results could, to a certain degree, be generalizable to countries where rock quality and bolting practice is similar to Sweden, which is commonly the case in areas where climbing is well-developed, and the rock frequently climbed. While some injury reports in the present study are from as early as 2008, the bolting practice and traditional equipment used have not changed considerably since, which mitigates this potential flaw.

The small sample size is a limitation of this study. The present study, however, did not intend to make a thorough description of all climbing related injuries occurring outdoors, but instead focus on the more severe injuries resulting in health care visits, which naturally limits the sample size. Other limitations of the study include the use of self-reported questionnaires, which always entail the risk of recall bias, and in this case also sampling bias. However, the use of medical records for verification of injuries and injury severity may mitigate this flaw to a certain degree. Furthermore, since the results of this study represent a Swedish setting, they may therefore not be applicable in places where a different environment or different climbing techniques are common.

To facilitate inter-study comparison, we suggest future studies to present results individually for each climbing discipline, separating outdoor and indoor climbing as well as clearly defining traumatic and overuse injuries. Studies using larger sample sizes and study methods of prospective nature are needed to reduce the risk of report bias and provide information regarding incidence rates of the different rock-climbing disciplines.

## Conclusion

Traumatic injuries sustained during outdoor climbing in Sweden were predominantly caused by falls and affected the lower extremities in all major outdoor climbing disciplines. Rope management errors as a cause of injury were common in sport climbing and in activity surrounding the climbing, indicating there is room for injury-preventing measures.

## Data Availability

The datasets used and/or analyzed during the current study are available from the corresponding author on reasonable request.
